# Contributions of neuroimaging in central poststroke pain: a review

**DOI:** 10.1055/s-0044-1789225

**Published:** 2024-08-31

**Authors:** Marcelo Delboni Lemos, Luciana Mendonça Barbosa, Daniel Ciampi de Andrade, Leandro Tavares Lucato

**Affiliations:** 1 Universidade de São Paulo, Faculdade de Medicina, Departamento de Radiologia, São Paulo SP, Brazil.; 2 Universidade de São Paulo, Faculdade de Medicina, Departamento de Neurologia, São Paulo SP, Brazil.

**Keywords:** Neuralgia, Chronic Pain, Stroke, Neuroimaging, Magnetic Resonance Imaging, Neuralgia, Dor Crônica, Acidente Vascular Cerebral, Neuroimagem, Imageamento por Ressonância Magnética

## Abstract

**Background** Central neuropathic poststroke pain (CNPSP) affects up to 12% of patients with stroke in general and up to 18% of patients with sensory deficits. This pain syndrome is often incapacitating and refractory to treatment. Brain computed tomography and magnetic resonance imaging (MRI) are widely used methods in the evaluation of CNPSP.

**Objective** The present study aims to review the role of neuroimaging methods in CNPSP.

**Methods** We performed a literature review of the main clinical aspects of CNPSP and the contribution of neuroimaging methods to study its pathophysiology, commonly damaged brain sites, and possible differential diagnoses. Lastly, we briefly mention how neuroimaging can contribute to the non-pharmacological CNPSP treatment. Additionally, we used a series of MRI from our institution to illustrate this review.

**Results** Imaging has been used to explain CNPSP pathogenesis based on spinothalamic pathway damage and connectome dysfunction. Imaging locations associated with CNPSP include the brainstem (mainly the dorsolateral medulla), thalamus (especially the ventral posterolateral/ventral posteromedial nuclei), cortical areas such as the posterior insula and the parietal operculum, and, more recently, the thalamocortical white matter in the posterior limb of the internal capsule. Imaging also brings the prospect of helping search for new targets for non-pharmacological treatments for CNPSP. Other neuropathic pain causes identified by imaging include syringomyelia, multiple sclerosis, and herniated intervertebral disc.

**Conclusion** Imaging is a valuable tool in the complimentary evaluation of CNPSP patients in clinical and research scenarios.

## Resumo

**Antecedentes** A dor neuropática central pós-acidente vascular cerebral (DNPAVC) afeta até 12% dos pacientes com AVC em geral e até 18% dos pacientes com déficits sensoriais. Essa síndrome dolorosa costuma ser incapacitante e refratária ao tratamento. A tomografia computadorizada e a ressonância magnética do cérebro são métodos amplamente utilizados na avaliação da DNPAVC.

**Objetivo** Este estudo tem como objetivo revisar o papel dos métodos de neuroimagem na DNPAVC.

**Métodos** Realizamos uma revisão da literatura sobre os principais aspectos clínicos da DNPAVC e a contribuição dos métodos de neuroimagem para estudar a fisiopatologia da DNPAVC, locais cerebrais comumente lesados na DNPAVC e possíveis diagnósticos diferenciais. Por fim, mencionamos brevemente como a neuroimagem pode contribuir no tratamento não farmacológico da DNPAVC. Além disso, utilizamos uma série de imagens de ressonância magnética da nossa instituição para ilustrar esta revisão.

**Resultados** Os exames de imagem têm sido usados para explicar a patogênese da DNPAVC com base no dano da via espinotalâmica e na disfunção do conectoma. Os locais de imagem associados à DNPAVC incluem o tronco cerebral (principalmente o bulbo dorsolateral), o tálamo (especialmente os núcleos ventral posterolateral/ventral posteromedial), áreas corticais como a ínsula posterior e o opérculo parietal e, mais recentemente, a substância branca tálamo-cortical no membro posterior da cápsula interna. Os exames de imagem também trazem a perspectiva de auxiliar na busca de novos alvos para tratamentos não farmacológicos para DNPAVC. Outras causas de dor neuropática identificadas por exames de imagem incluem siringomielia, esclerose múltipla e hérnia de disco intervertebral.

**Conclusão** Os exames de imagem são uma ferramenta valiosa na avaliação complementar de pacientes com DNPAVC em cenários clínicos e de pesquisa.

## INTRODUCTION

Stroke is the second most common cause of death and a major cause of morbidity worldwide. 1 Besides disability related to aphasia, reduced mobility, and depression, up to 55% of stroke survivors will develop chronic poststroke pain (PSP). 2 This condition includes a range of pain syndromes, with distinct clinical manifestations, mechanisms, and treatments, such as musculoskeletal pain, spasticity-related pain, headaches, complex regional pain syndrome, and central neuropathic pain (that is, central poststroke pain [CPSP]). 3 4

Central poststroke pain occurs in up to 12% of patients with stroke in general 2 and up to 18% in patients with sensory deficits. 2 This pain syndrome, whose mechanisms are not fully understood, has challenging clinical management. Patients often have lifelong pain symptoms with poor response to current therapies. There are commonly associated sleep and mood disorders that impair rehabilitation and decrease overall quality of life. 5 6

Since the beginning of the twentieth century, clinical and pathological CPSP case studies have found commonly damaged brain areas in these patients, particularly the thalamus, 7 the parietal cortex, 8 and, later, the posterior insula. 9 Currently, many authors defend that CPSP emerges from lesions along the spinothalamocortical afferent system, although this is not an independent factor and other variables in addition to the stroke are also probably necessary. 10

Modern imaging techniques have largely contributed to confirm clinical and surgical evidence that damage to certain brain areas is related to CPSP. 11 12 13 Neuroimaging is widely used to support clinical diagnosis, 14 help the differential diagnosis of uncertain cases, 15 and it can work as a tool to investigate the mechanisms behind the pathophysiology. 16 The accurate topographical characterization of which brain areas are involved in this syndrome has potential benefits in clinical practice since imaging methods can help unveil its mechanisms, develop non-pharmacological treatments and identify patients at greater risk of developing CPSP, allowing the possibility of early or prophylactic interventions. Therefore, imaging methods may contribute to a better allocation of patients in clinical trials and prospective studies could also test the influence of prophylactic treatment in stroke victims with an increased chance to develop CPSP based on risk stratification through imaging.

The objective of the present review is to demonstrate the possible contributions of neuroimaging in the diagnosis, pathophysiology and, particularly, the radiological findings in patients with CPSP. Differential diagnosis and image contributions to the treatment field will also be discussed.

## CLINICAL CHARACTERISTICS

Although variable, most patients with PSP experience pain from 3 to 6 months after stroke. 17 Symptoms and their clinical categorization are complex and not uniform, and generally, PSP is considered an umbrella term comprising pain syndromes with different mechanisms including neuropathic pain (CPSP) and non-neuropathic pain syndromes: headaches, musculoskeletal pain, shoulder pain, and painful spasticity. 2 These groups are not mutually exclusive nor comprehensive; therefore, patients can experience both non-neuropathic and neuropathic types of pain 18 as well as any combination of PSP with other types of pain not contemplated by this classification, not to mention preexisting chronic pain related to other conditions.

Patients with CPSP typically have pain corresponding to the location of the vascular lesion, most commonly the body side contralateral to the infarcted area and refer classic neuropathic pain descriptors. In this sense, features do not differ from other central or peripheral neuropathic pain etiologies. 19 Pain is frequently described as “burning,” “freezing,” “electric,” and “stabbing,” among others. It can be spontaneous or evoked by non-painful stimuli such as touch and cold. 4 Physical examination demonstrates somatosensory dysfunction in the affected body area. 20 Different combinations of negative (hyposensitivity to cold or touch, mechanical hypoalgesia) and positive signs (cold or touch hyperesthesia, mechanical hyperalgesia, cold or mechanical allodynia, and hyperpathia) can be observed. 4 Signs of spinothalamic dysfunction with sensory changes related to the pain or temperature perception, especially for cold, are frequently noted. 4 20

The non-neuropathic poststroke type of pain comprises musculoskeletal pain 21 (such as painful spasticity, shoulder, and back pain) and tension-type headache. 22 Despite also occurring after stroke, these painful syndromes present a mix of mechanisms, distinct from neuropathic pain, most of which are related to nociceptive stimuli and central and peripheral sensitization. It is thought to arise from weakness, biomechanical changes, malposition, subluxation, mechanical overload, immobility, and joint and proprioception impairment after brain injury. 17

The diagnosis of neuropathic pain and other pain syndromes is predominantly clinical and often challenging. 2 23 As previously mentioned, many patients present more than one pain syndrome. The differentiation between these syndromes is fundamental to guide the patient's therapeutic approach, which must be performed on an individual basis and based on their pain mechanisms. Detailed clinical assessment through pain descriptors and physical examination can help differentiate these syndromes, especially with a focus on pain descriptors and the presence of cold thermal hypoesthesia and allodynia. 4

## PATHOPHYSIOLOGY OF CPSP

The mechanisms involved in CPSP are not elucidated. Moreover, the understanding of the physiology of chronic pain in general is still largely unclear. 24 Unlike other primary senses such as vision and audition, there are many gaps in the understanding of principles of pain processing, let alone the subjective component involved in pain perception and modulation.

Apart from the discriminative sensory information limb carried by the spinothalamic pathway and its thalamocortical projections, pain also has an autonomic response component, which can be explained by the extensive spinothalamic synapses with homeostatic integration sites in the spinal cord (preganglionic sympathetic neurons in the lateral column of the thoracolumbar spinal cord), the brainstem (such as the ventrolateral medulla, parabrachial nuclei in the pons and periaqueductal gray in the midbrain), and the hypothalamus. 25 In addition, pain also has an inherent emotional and cognitive component, evident in the cortical projections to the anterior insula, prefrontal cortex, and cortical limbic regions, such as the amygdala and anterior cingulate cortex. 26 With such diffuse projections of the nociceptive stimuli, some authors believe there is no single pain perception hub, as multiple cortical areas are involved in the integration and analysis of the pain experience, in a complex process typically described as multidimensional. 27

The proposed mechanisms involved in CPSP should reflect the clinical manifestations of patients, notably the chronic nature, the spontaneous characteristics of pain, and the exaggerated sensation evoked by stimuli. Some possible theories and their respective imaging contributions are described below.

### Spinothalamic (ST) pathway damage

Clinical evidence shows that patients with CPSP commonly have dysfunction of the ST pathway. 28 There is hypoesthesia to pinprick and cold, and pain is localized in areas in which sensation is lost or disrupted. In addition, imaging methods often demonstrate stroke lesions in various brain structures related to the ST pathway. 29 Therefore, the first theories focused on the damage to the ST pathway and, consequently, the distorted interactions between different sensory modalities. One such theory regards the imbalance between the thermal and pain sensations contributing to CPSP, according to the thermal disinhibition hypothesis. 30 Damage of the pathway component that transmits thermal information, which normally inhibits fibers that carry pain signals to the cortex, allows emergence of pain through its disinhibition. This hypothesis was tested in experimental models and supported by imaging studies. A meta-analysis with 23 functional positron emission tomography (PET) and magnetic resonance imaging (MRI) scans showed that brain activation due to thermal stimuli is coincident with pain and noxious stimuli. 31 Functional MRI (fMRI) data also reinforced the importance of the posterior insula in temperature perception, with somatotopic maps for noxious heat and cold. 32 However, a fMRI study in patients with syringomyelia, which characteristically have ST dysfunction and may present with central pain, failed to demonstrate a direct relationship between the sensory loss type and the presence or intensity of neuropathic pain. 33

### Connectome dysfunction: structural and functional connectivity

The comprehensive pathogenesis of CPSP evolved along the emerging concept that physiologic pain results from the coordinated activity of different integrated brain networks, 34 rather than a simple on/off phenomenon. Therefore, CPSP has been more recently thought of as a reorganization disorder, in which “pain matrix” structures suffer neuroplasticity and adapt to the new, lesioned reality that results in pathologic functioning. 35 36 This is in line with the chronologic evolution of CPSP, which has an adaptive nature and progressive onset.

Different neuroimaging studies support these ideas. Neuroplasticity and reorganization of critical hubs in the pain network were demonstrated in a Japanese study with structural MRI and voxel-based morphometry (VBM) using a monkey model of CPSP. 37 After inducing a lesion in the ventral posterolateral nucleus of the thalamus, the authors found significantly reduced gray matter volume in the anterior and posterior insulas and in the somatosensory cortex, confirmed by histological analysis. Similar findings were also observed in another study with VBM, which compared 45 poststroke patients, 23 of them with PSP and 22 without pain; in addition to somatosensory and insular cortex reduced volume, there was also decreased gray matter in the prefrontal and orbitofrontal cortex, areas related to the affective component of pain. 38 Functional imaging with both fMRI and PET has also shown activity in a broad network of brain sites during chronic pain after stroke, supporting the idea of multilevel, brain-wide interactions to produce CPSP. A combined PET and fMRI case study in a patient with CPSP after a parietal stroke reported increased activity in the insula/somatosensory cortex during allodynia, but lacked other brain areas generally active in normal individuals with pain, which may suggest that those with PSP experience pain differently than non-stroke individuals with chronic pain. 39 Magnetoencephalography (MEG) was also used in CPSP patients to evaluate brain activity in the presence of anticipation of pain. The thought of imminent pain produced parietal and frontal cortex activations in the healthy hemisphere but decreased responses in the unaffected side. Authors theorized that these results could represent a downward modulation in the lesioned hemisphere to adapt to the constant firing of pain signals produced by the dysfunctional pain matrix. 40

Structural neuroimaging studies have also demonstrated the importance of damage to the thalamic pulvinar in CPSP. 13 29 41 This nucleus does not receive ascending spinothalamic inputs but rather acts as an important association hub with exclusive cortico-cortical connections (to and from the thalamus) engaged in the trans-thalamic routing of cortical networks. 42 This dynamic synchronized activity of distant cortical regions, partly mediated by the thalamus, is thought to sustain the subjective sensory experience and conscious awareness of afferent inputs, including pain. 43 44 Abnormal function of these networks is demonstrated in chronic pain states by electrophysiological studies such as corticothalamic dysrhythmia. 45 Thus, disruption of the multi-regional cortical network via the trans-thalamic route, whether due to damage of cortico-thalamic connections or to the medial pulvinar, may also contribute to thalamocortical desynchronization in PSP.

### EPIC model

The emergence of neuropathic pain after thalamocortical disconnection in stroke may also be explained using the recently proposed Embodied Predictive Interoception Coding (EPIC) model, a general theory regarding interoception and its processing in the brain. 46 According to this model, the posterior insula receives and amplifies ascending sensory signals arriving from the thalamus and projects this information to other cortical areas, such as the anterior insular cortex. In turn, the anterior insular cortex sends sensory predictions to the posterior insula, where neurons in the granular cortex compare these predictions with the actual incoming ascending inputs. Prediction error would be measured and then signaled back to the anterior insula, leading to dynamic changes in sensory gain and behavior modulation to meet prediction with the real sensory experience and, ultimately, maintain homeostasis. 26 In CPSP, the deafferentation of the thalamic projections, either via the thalamic route or white matter interruption, would lead to failure to confront the (distorted) signals from the internal milieu against the interoceptive state predicted by the anterior insula. Prediction error measurements would be jeopardized, and the salience system would lose its precision as a tool to modulate incoming sensory inputs in an adaptive manner. The end result of these processes would be a hyper-activated salience system leading to excessive somatosensory gain (allodynia, hyperalgesia and spontaneous pain), demonstrated in functional neuroimaging studies as a hyperactive insula typical of neuropathic pain conditions and pain-evoked cortical responses. 47 48 49

## DIAGNOSIS

The diagnosis of CPSP can be challenging. There are few standardized diagnostic criteria, the clinical picture can be variable, and patients may experience overlapping types of chronic pain, making the differentiation difficult.

History and physical examination should be assessed with imaging studies and sensory examinations. One proposed diagnostic criterion for CPSP include mandatory and supportive criteria 2 19 : mandatory criteria are pain within an area of the body corresponding to the central nervous system lesion; history suggestive of stroke and onset of pain after stroke onset; confirmation of stroke by imaging or negative/positive sensory signs in the affected area; and exclusion of other pain causes. Supportive criteria include pain not related to movement, inflammation, or other local tissue damage; typical neuropathic pain descriptors; and the presence of allodynia or dysesthesia to touch or cold. 2

Both CT and MRI can be performed to confirm the history of stroke. Computed tomography scans are useful to diagnose stroke and evaluate its extension, particularly in the emergency setting, but may be false negative if the image is acquired too early or the lesion is subtle. Magnetic resonance imaging is preferable due to its higher sensitivity to small lesions, increased spatial resolution, and better characterization of differential diagnoses. However, it is also less available, more expensive and patients may deal with safety and compatibility issues. The image should assess the type of stroke (ischemic or hemorrhagic), lesion topography and size, and potential etiologic mechanism (with or without angiographic studies). Typical brain regions associated with CPSP are demonstrated below. As stated, imaging can also be used to evaluate other causes of neuropathic pain not related to stroke, such as multiple sclerosis, syringomyelia, herniated intervertebral disc, and others.

## IMAGING LOCATIONS ASSOCIATED WITH CPSP

### Brainstem

The lateral medullary infarct and its resultant clinical Wallenberg syndrome is the most common brainstem stroke associated with CPSP. In a study with 63 patients with lateral medullary infarct, 25% developed CPSP. 50 These infarcts generally produce severe pain ipsilateral in the face, particularly in the peri-orbital and cheek regions. Facial pain can be isolated or associated with contralateral limb and extremity pain. Interestingly, a study also observed that quantitative sensory testing of the contralateral trigeminal territories in the face were normal in cases of CPSP; therefore, crossed trigeminal fibers which travel in the ventral trigeminothalamic (TT) tract are probably spared in CPSP patients. 51 These TT fibers are located in the medial medulla, adjacent to the reticular formation, which receives extensive ST input and produces a diffuse spinoreticolothalamic output. Therefore, clinical evidence points to the occurrence of CPSP in medullary infarcts after damage of the ST tract in the lateral medulla, concomitant with sparing of the spinoreticolothalamic fibers and, indirectly, TT fibers, both located in the medial medulla. 51

### Thalamus

**Figure 1:**
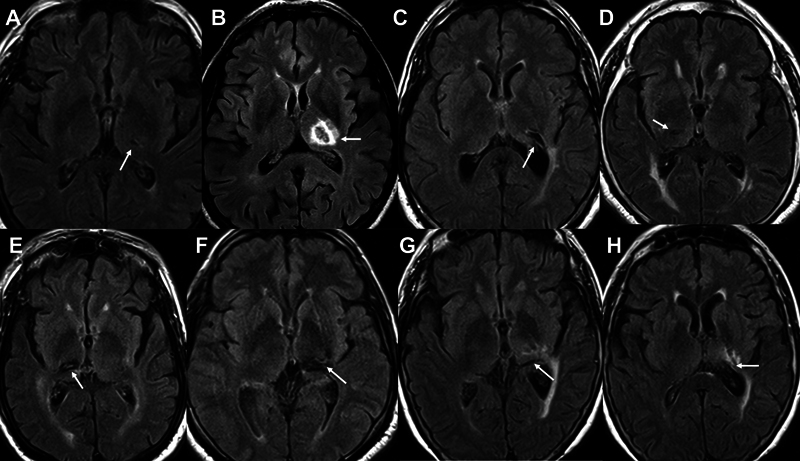
Axial fluid-attenuated inversion recovery (FLAIR) magnetic resonance imaging (MRI) scans of different patients with central poststroke pain (CPSP) and thalamic infarcts illustrating the diversity of lesions able to cause neuropathic pain. ( **A** ) Small lacunar infarct on the centrolateral left thalamus. ( **B** ) Large infarct encompassing most of the left thalamus. ( **C** ) Chronic lacunar infarct in the posterolateral aspect of the left thalamus. ( **D** ) Small lacunar infarct in the centrolateral right thalamus. ( **E** ) Hemorrhagic infarct in the posterolateral aspect of the right thalamus. ( **F** ) Chronic hemorrhagic stroke in the pulvinar and posterolateral aspect of the left thalamus. Images of the same patient showing a chronic stroke in the pulvinar and posterolateral aspect of the left thalamus ( **G** ) as well as gliosis of the adjacent white matter ( **H** ).

The thalamus is the most classic brain site involved in CPSP ( Figure 1 ). Most studies with imaging in CPSP demonstrate stroke topography centered in the ventral posterolateral/ventral posteromedial (VPL/VPM) nuclei. 11 13 41 52 These are pivotal ST/TT targets and important relay points of nociceptive inputs to the cortex, as traditionally demonstrated in experimental studies in cat and primates. 53 54 However, thalamic targets of the ST/TT were also questioned in the literature. Craig et al. used a high-resolution anterograde tracer to demonstrate that, in primates, spinothalamic neurons project to a distinct dedicated nucleus named the posterior part of the ventromedial nucleus (VMpo). 55 They also characterized the microscopic features of this nucleus in the human brain and its anatomic relationship with other thalamic nuclei. 56 However, the existence of the VMpo has been questioned by some authors 57 and was not confidently shown in neuroimaging studies to date.

### Cortical areas

**Figure 2:**
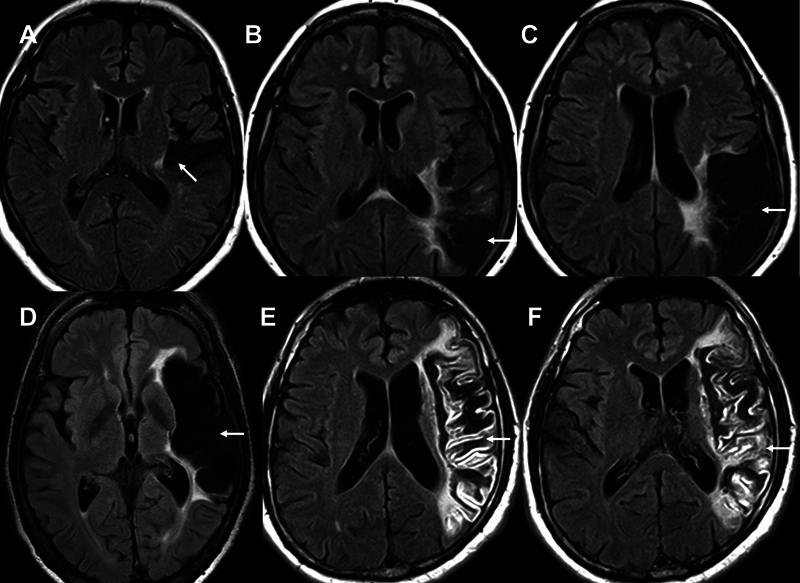
Axial FLAIR MRI scans showing cortical stroke lesions. All patients have neuropathic poststroke pain. ( **A** ) Infarct of the left posterior insula and parietal operculum area. ( **B,C** ) Images of the same patient depicting a large infarct in the territory of branches of the inferior division of the left middle cerebral artery, including the posterior insula and the medial parietal operculum areas. ( **D** ) Large sequelae including almost the full territory of the cortical branches of the left middle cerebral artery. ( **E,F** ) Images of the same patient showing a late subacute infarct of the full territory of the left middle cerebral artery.

There is enough evidence to attribute the cortical representation of the thalamic relay of nociceptive input to the posterior insula and the medial parietal operculum (PIMO), a distinct region from the remainder of the somatosensory stimuli, usually located in the somatosensory (SI) cortex. 10 57 In an experimental study in primates, Dum et al. 58 infected first order dorsal horn neurons related to the ST tract with herpes virus and used anterograde transneuronal transport to follow these particles to third-order cortical neurons. They estimated that ∼ 40% of ST tract fibers terminate in the granular or posterior insula, 30% in the medial parietal operculum, 25% in the mid-cingulate cortex and only ∼ 5% in the primary somatosensory cortex. Neuroimaging studies in CPSP constantly report stroke occurring in the PIMO location 29 59 ( Figure 2 ).

The anterior cingulate cortex (ACC) is also consistently active in imaging studies of subjects exposed to painful stimuli, 60 61 as the ACC is part of the limbic system, thought to be involved in the emotional and cognitive processing of pain. However, there is not enough evidence that this brain area is damaged in CPSP patients.

### Thalamocortical white matter

**Figure 3:**
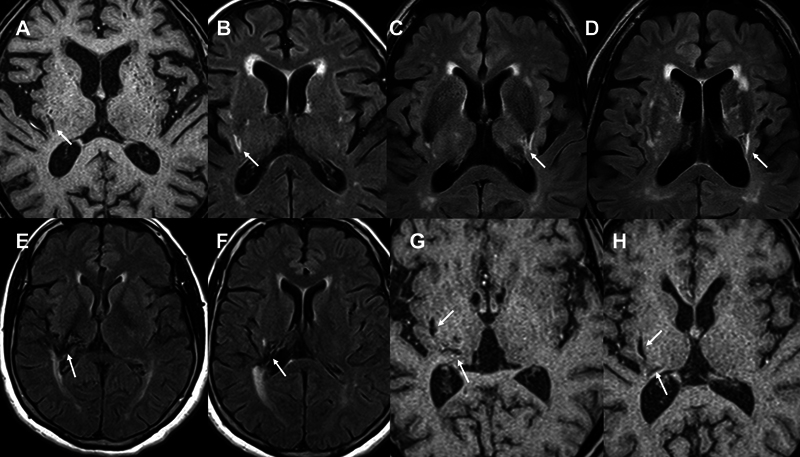
Magnetic resonance imaging scans demonstrating lesions in the thalamocortical white matter topography in patients with CPSP. These lesions may occur isolated in the posterior limb of the internal capsule, retroputaminal white matter, or subinsular area; or associated with posterolateral thalamic lesions. Axial images of the same patient showing an isolated lacunar infarct in the right subinsular and retroputaminal area (T1- weighted image, **A** ) with adjacent gliosis (FLAIR image, **B** ). Axial FLAIR images of the same patient depicting a chronic left lacunar infarct in the retroputaminal area ( **C** ) with cranial extension into the subinsular area ( **D** ). Axial FLAIR images of the same patient showing a sequela of hemorrhagic stroke in the posterior limb of the internal capsule ( **E** ) with extension into the subinsular area ( **F** ). ( **G,H** ) Axial T1-weighted images of the same patient showing lacunae in the posterolateral thalamus and subinsular area.

In addition to the classic brain areas involved in CPSP, some studies have found that these patients have damage in the white matter adjacent to the thalamus, in the posterior limb of the internal capsule, particularly in the retrolenticular portion. 29 41 62 One study showed that this region damage is among the highest odds-ratio to the occurrence of CPSP. 41 Connections between the thalamus and the posterior insula/parietal operculum were demonstrated in experimental studies with radiotracers in monkeys, notably including the pulvinar and the posterior thalamus with the granular insula and retro-insular areas. 54 Similar connections were identified in humans in a MRI tractography study. 63 Lesions in the white matter deep to the caudal insula and opercular region were described in a CT and MRI case series of 20 patients with PSP. 62 Kim 62 and Landerholm et al. 64 theorized that these lesions in the posterior putamen and posterior limb of the internal capsule in CPSP were potentially related to the interruption of thalamocortical pathways. Examples of clinically observed infarcts in the thalamocortical white matter topography are illustrated in Figure 3 .

Although less often debated in the literature, white matter interruption may be as important as classic gray matter damage in the emergence of CPSP. At least in a subset of these patients, neuropathic pain could emerge as a disconnection syndrome, a concept observed in conduction aphasia and apraxias in which cortical neurologic dysfunction may be reproduced by damage in associative structures of interconnected networks. 65

## DIFFERENTIAL DIAGNOSIS: OTHER NEUROPATHIC PAIN CAUSES IDENTIFIED BY IMAGING

### Syringomyelia

**Figure 4:**
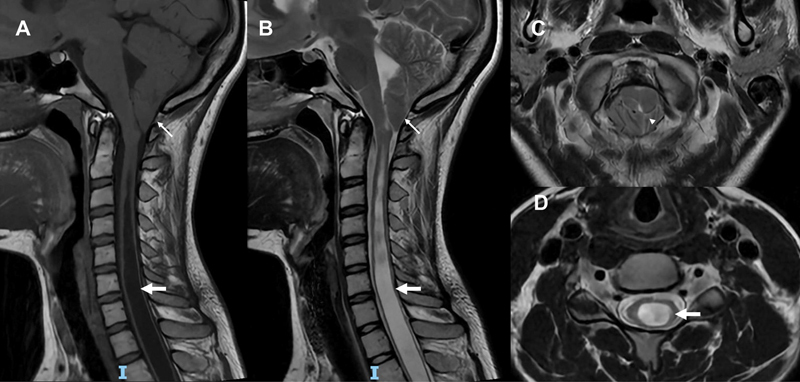
Magnetic resonance imaging scans of a patient with neuropathic upper limb pain showing Chiari I abnormality and syringomyelia. Sagittal T1-weighted ( **A** ) and T2-weighted ( **B** ) images depicting caudal tonsillar herniation below the foramen magnum (arrows) and extensive syringohydromyelia (thick arrows). ( **C** ) Axial T2-weighted image shows narrowing of the craniovertebral junction; the cisterna magna is obliterated by the cerebellar tonsils (arrowhead). ( **D** ) Axial T2-weighted image showing that the syringohydromyelia is slightly shifted toward the posterior quadrants in the cross-section of the spinal cord (arrow).

Neuropathic pain is not uncommon in syringomyelic patients, as well as in other types of injury to the spinal cord, and its clinical characteristics may be similar to patients with CPSP and upper limb pain 66 ( Figure 4 ). Conventional MRI demonstrated that pain is more prevalent in patients in which the spinal cavity extended into the dorsolateral cross-sectional quadrant of the spinal cord, probably secondary to the involvement of afferent nociceptive stimuli entry into the dorsal horns and its spinothalamic output. 67 Diffusion tensor MRI was also able to demonstrate greater microstructural white matter damage in the spinal cord (assessed by fractional anisotropy) in syringomyelic patients with neuropathic pain with higher average daily pain intensities. 68

### Multiple sclerosis

Multiple sclerosis (MS) and chronic pain are highly comorbid. 69 Patients may experience both musculoskeletal and/or neuropathic pain, as in PSP, and pain is thought to arise secondary to both demyelination/neuroinflammation and brain lesion mechanisms. 69 As multiple lesions are frequent throughout the neuraxis, it is difficult to assign a specific lesion as a culprit for the cause of pain, and candidate lesions are often suggested based on anatomical plausibility. A systematic review of pain in MS and its neuroradiological correlates showed that patients with facial pain and trigeminal neuralgia have predominantly brainstem and trigeminal nuclei damage compared with other types of pain. 70

### Herniated intervertebral disc

**Figure 5:**
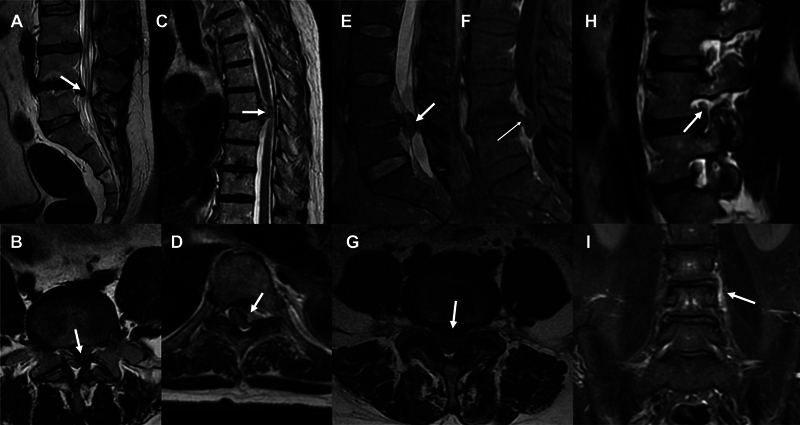
Variety of intervertebral disc hernias detected by MRI in patients with neuropathic pain. Sagittal ( **A** ) and axial ( **B** ) T2-weighted images of the same patient showing L4-L5 disc hernia with compression of the left descending L4 and L5 nerve roots inside the thecal sac (arrows). Sagittal ( **C** ) and axial ( **D** ) T2-weighted images of the same patient showing mid thoracic disc hernia with compression of the spinal cord (arrows). Sagittal ( **E,F** ) and axial ( **G** ) images of the same patient showing intervertebral disc extrusion (T2 -weighted image in **E** and **G** ) with radicular conflict in the thecal sac (arrows). The postcontrast T1-weighted image ( **F** ) shows the exact point of annulus fibrosus rupture that led to discal content extravasation (arrow). Sagittal ( **H** ) and coronal ( **I** ) T2-weighted images of the same patient showing left foraminal L3-L4 disc hernia (arrow) abutting the L3 root ( **H** ). Despite seemingly mild contact, these findings were compatible with the dermatome symptomatology and were associated with edema of the left L3 nerve root (arrow in **I** ).

Radiculopathy secondary to a herniated intervertebral disc is one of the most classic forms of neuropathic pain. Although often originated from the peripheral nervous system, herniated discs may produce symptoms akin to those observed in central neuropathic pain. 71 Magnetic resonance imaging can demonstrate which nerve roots are involved, the anatomic relationship of the root with the disk hernia, the degree of neural compression, and edema of the surrounding tissues, and is the method of choice to evaluate response to surgical treatment. Examples of intervertebral disc hernias detected by MRI are illustrated in Figure 5 .

## TREATMENT

Central poststroke pain remains a challenging condition to treat. There are few randomized clinical trials on CPSP to guide novel and current therapies. The first line of treatment for the condition is pharmacologic, with tricyclic antidepressants, duloxetine, and gabapentine. 72 However, CPSP is often refractory to medication, resulting in increased dosages, with consequent increased adverse effects, producing only modest decreases in pain in a limited subset of patients. 2 As such, alternative methods of pain control are needed.

Neuromodulation techniques are frequently used in the treatment of refractory chronic neuropathic pain, including CPSP. These methods deliver targeted stimuli (mostly electrical or magnetic) to the nervous system with the goal to alter neural activity and induce functional and structural changes (neuroplasticity). 73 The process may be executed with invasive and noninvasive modalities, and neuroimaging is frequently used to guide treatment with anatomic precision. In CPSP, the most used invasive techniques are motor cortex stimulation and deep brain stimulation (DBS). 73

In motor cortex stimulation, the most studied and used technique, electrodes are installed superficial to the motor cortex and fibers underneath, then electrical stimulation is applied through a pulse generator. Prior to surgery, fMRI may be acquired with motor tasks to improve motor cortex mapping in addition to neuronavigation techniques. 74 Great efforts have been made in finding new targets, and neuroimage studies can assist in this task and perhaps help selecting patients for specific targets according to the compromised territory.

Deep brain stimulation consists in delivering electrical impulses to specific brain targets by surgically implanted electrodes. The mechanism of action is not well understood, but most authors believe the effects are due to the modulation of dysfunctional neural networks. 75 Deep brain stimulation targets as a treatment for CPSP include the ventral posterior medial/ventral posterior lateral nuclei, centromedian thalamic nuclei, internal capsule, periaqueductal gray matter, and the anterior cingulate cortex, and bilateral stimulation is often preferred. 76 To locate brain targets, high-resolution structural MRI is used in association with stereotactic methods. Probabilistic tractography may also be used to improve mapping through the analysis of structural connectivity. 77

There are other neuromodulation techniques used in CPSP that do not require surgery (noninvasive), the most used being repetitive transcranial magnetic stimulation (rTMS). This technique delivers a magnetic field in a train of repetitive pulses that can induce electrical changes in neurons and modulate activity in underlying brain structures. 78 79 Structural MRI is also used to guide transcranial magnetic stimulation (TMS), although this treatment needs a rougher idea of the targeted brain site than the invasive methods. 79

In conclusion, CPSP is a syndrome with lifelong symptoms and major impact on quality of life. Diagnosis is based on clinical criteria and supported by imaging evidence of stroke. Imaging is also used in the clinical scenario as a supplementary method to identify specific brain structures commonly damaged in CPSP and may also complement the differential diagnosis of other chronic neuropathic pain conditions. Techniques such as fMRI, PET, VBM, and lesion-symptom mapping are used in research as tools to investigate neuroplasticity, dysfunction of the “pain matrix,” and other hypothesized mechanisms behind the syndrome. Despite the advances in understanding the pathogenesis and anatomy of CPSP, it remains unknown which patients will develop central neuropathic pain after stroke, as the target lesions alone are not sufficient to trigger the syndrome and other variables are also necessary. Moreover, response to current therapies remains poor, regardless of the increased anatomic precision to deliver treatment. It is hoped that future studies will address these questions regarding this challenging pain syndrome.
